# Cultural Adaptation of the SADL (Satisfaction with Amplification in Daily Life) questionaire for Brazilian Portuguese

**DOI:** 10.1590/S1808-86942011000500005

**Published:** 2015-10-22

**Authors:** Maria Fernanda Capoani Garcia Mondelli, Fabiani Figueiredo Magalhães, José Roberto Pereira Lauris

**Affiliations:** 1 PhD, Professor at the Dentistry School - Bauru-USP; 2 MSc in Speech and Hearing Therapy - Dentistry School of Bauru - University of São Paulo., Scholarship at the Technical Training by FAPESP; 3 Associate Professor - Department of Odontopediatrics, Orthodontics and Collective Health - Dentistry School of Bauru - University of São Paulo, FOB/USP

**Keywords:** adaptation, hearing loss, questionnaires

## Abstract

**Abstract:**

There are some motivational processes associated with the use of an Individual Sound Amplification Device (ISAD): acceptance, benefit and satisfaction.

**Objective:**

To make a cultural adaptation of the SADL (Satisfaction with Amplification in Daily Life) questionnaire to use with the Brazilian population; to assess its reproducibility and to describe its results in patients fit with an ISAD.

**Materials and Methods:**

Clinical study. Translation and cultural adaptation of the questionnaire; translation from English into Portuguese and linguistic adaptation; review of the grammatical and idiomatic equivalence; evaluation of the inter and intra-researcher assessment. There were 30 ISAD users older than 18 years of age participating in the study (mean: 66.36 years); 19 men (63%); 11 women (37%).

**Results:**

The participants reported greater satisfaction (mean) for questions: 1 (6.35); 3 (6.85); 5 (6.10); 6 (6.80); 8 (6.33); 9 (6.80); 11 (6.16); 12 (6.93) and 15 (6.52). Less satisfaction: question 4 (3.16); 7 (3.41) and 13 (2.83). The comparison of each question with the first and second application of the questionnaire did not present statistically significant results, thus yielding good reproducibility. There was a greater satisfaction for subscales: Positive Effect (6.50) and Services and Costs (6.26) and less satisfaction for Negative Factors (4.73).

**Conclusion:**

It was possible to adapt the questionnaire for the Brazilian population.

## INTRODUCTION

Hearing is fundamental for human life; it plays an important role in society, because it is the very basis of human communication development. Individuals with hearing impairment may suffer severe loss in their social, psychological and professional lives, also having feelings of insecurity, fear, depression, isolation and also family tensions because of the lack of attention affecting those with hearing disorders.

The problems caused by sensorial deprivation may be minimized with the use of an Individual Sound Amplification Device (ISAD), which helps rescue speech sound perception, and also that of environmental sounds, promoting an improvement in communication skills^1^.

In the year 2000, the Ministry of Health (MH) approved Ordinance SAS#43^2^,^2^, focused on the social importance of hearing loss and on the need of broadening the provision of ISADs to patients from the Public Health Care System (SUS). Nonetheless, the follow up and the hearing rehabilitation programs for these individuals did not grow at the same pace, making hearing aids often times underestimated and/or underutilized. With the aim of enhancing hearing loss care, in 2004, the National Policy of Hearing Health Care was approved, by means of Ordinances GM2073 and SAS58^7^,^3^, and according to this policy, they encompassed actions to impact on the natural history of hearing loss through integral actions for health promotion, specific protection, treatment (involving the provision of hearing aids when indicated) and auditory rehabilitation.

Thus, the Individual Sound Amplification Device provision program proposes care for children and adults with hearing impairment concerning the functional use of their auditory reserve, enabling the use of residual hearing and helping the individual interact with society.

During the process of advising hearing impaired individuals, we must be concerned with three motivational processes associated with the use of a sound amplification device: acceptance, benefit and satisfaction^4^. Satisfaction is built according to the individual's own impressions. Thus, it is clear that without acceptance there will never be satisfaction, and not every acceptance and benefit associated with the device is enough to guarantee satisfaction. While the benefit can be shown by these objective tests, satisfaction is a very personal judgment on the hearing aid after a given time of use^5^.

We can say that the checking procedures, such as functional gain and insertion measures, are not enough to assess the patient's satisfaction with the hearing aid in daily communication situations. There was a growing interest in developing validation procedures which would allow the assessment of the user's benefit outside the clinical setting, made up of self-assessment questionnaires^6^.

Self-assessment is a simple, fast and efficient procedure, which enables the evaluation of the individual during his/her fitting process. This procedure enables the comparison between different devices and/or calibrations, as well as the assessment of the benefits achieved with the hearing aid along time, enabling the user to recognize the advantages provided with the fitting of an individual sound amplification device (ISAD) in relation to his-her hearing difficulties and psychosocial disadvantages. Thus, by means of questionnaires which enable measuring these auditory difficulties or handicaps, it is then possible to optimize the time necessary for one to adapt to sound amplification^5^.

Satisfaction is the measure of the auditory rehabilitation outcome, which aims at encompassing the most complete constellation of factors which are needed for the final result, since the variable of interest is the patient's opinion and it is not associated only with the ISAD performance, depending solely on the person's perceptions and behaviors^7^,^8^.

There are numerous assessment tools based on scales to assess the individual's level of satisfaction, because there are many factors impacting different dimensions associated with ISAD use^5^.

In Brazil, some self-assessment questionnaires, the APHAB (*Abbreviated Profile of Hearing Aid Benefit*), the HHIE (*Hearing Inventory for the Elderly*) and the HHIA (*Hearing Handicap Inventory for Adults*), which were translated and adapted to our country settings, are used to investigate the level of user satisfaction, the benefits obtained from the use of hearing aids and the reduction in hearing impairment with the use of the ISAD, and others which aimed at comparing the benefits of different technologies and to check the hearing aid fitting by means of objective and subjective measures^6^, ^7^, ^8^, ^9^.

The satisfaction with the use of an individual sound amplification device in daily life has also been studied by means of the *Satisfaction with Amplification in Daily Life* - SADL questionnaire, developed^7^ and evaluated by researchers^8^,^10^,^11^.

This tool was created in order to assess the user satisfaction with ISADs, quantifying it using four subscales: Positive Effects, Costs and Services, Negative Factors and Personal Image.

Most of the questionnaires analyze the individual's performance in different communication settings, or they try to obtain information on the disadvantage hearing loss can bring about. Others are also employed in the onset of the ISAD selection and fitting process in order to estimate its success, besides providing data which could guide the fitting. Therefore, these are subjective and qualitative measures which could help us understand user satisfaction concerning hearing aids and their day-to-day communication difficulties^12^.

The SADL has been used in some studies carried out in Brazil; nonetheless, its translation into Brazilian Portuguese is yet to be validated^13^.

As previously mentioned, the present paper aimed at making a cultural adaptation of the SADL to be used with the Brazilian population, to assess the SADL reproducibility and describe the results from the application of such questionnaire in patients fit with ISADs.

## MATERIALS AND METHODS

This study was only carried out after it was approved by the Ethics in Research Committee, with protocol number 074/2008 and patient's consent to participate in the study and to publish the data obtained.

## SERIES

We assessed 30 patients older than 18 years, of both genders, fit with an individual sound amplification device. The individuals had understanding and auditory thresholds which enabled the application of the SADL questionnaire.

### Tools and procedures

**Tool**: The translation and cultural adaptation of the SADL questionnaire followed the stages indicated by the authors^14^ which included the translation from English into Portuguese and linguistic adaptation, idiomatic and grammar equivalence review, and cultural adaptation. Moreover, we assessed the inter and intra-researcher reproducibility.

The questionnaire has 15 questions, broken down into four subscales: Positive Effects (6 items associated with the acoustic and psychological benefits), Service and Costs (3 items associated with professional competence, price of the product and technical assistance), Negative Factors (3 items associated with the amplification of environmental noise, feedback and the use of telephone) and personal image (3 items associated with cosmetic factors and the very stigma associated with using an ISAD) (Attachment 1).

Considering the 15 items present in the SADL, in 11 of them the score provided by the individuals coincided with the scoring scale and in the other 4 items (questions 2, 4, 7 and 13) there was an inverse relation between the score and the scale (in other words, in these cases score 1 receives 7 points and expresses a greater satisfaction). The higher the numeric results obtained by the mean values of the answers from each subscale, the greater the individual satisfaction. Questions 1,3,5,6,9 and 10 belong to the Positive Effects subscale; questions 2,7 and 11 belong to the Negative Factors subscale; questions 4,8 and 13 belong to the Personal Image subscale; and questions 12,14 and 15 belong to the Service and Costs subscale.

### Procedure

*Translation from English into Portuguese and linguistic adaptation*

The questionnaire was handed out to three English teachers-translators/interpreters, who did not know each other and had not seen the questionnaire before, in order to secretly and individually produce a Portuguese version of it. This procedure was carried out with the aim of creating three independent translations of the SADL. The reviser group was made up of three speech and hearing therapists (Brazilians, fluent in English), who analyzed the three resulting documents and, by consensus, reduced the differences found in the translations, choosing the best words and expressions used to translate all the questions, adapting the text to the Brazilian Culture. This stage was based on choosing the best translation for the questions and the modification by approximation of the most adequate terms, chosen to enable full understanding by the Brazilian population. Thus, we obtained and new and single questionnaire which was called *Brazilian Satisfaction With Amplification in Daily Life* Questionnaire, or Brazilian SADL (Attachment 2).

*Review of the idiomatic and grammar equivalence* Following, a copy of the Brazilian SADL was sent to three other Portuguese-English translators with the same language expertise of the first ones. These translators, without knowledge of the original text, translated it back into English. We did not allow these new translators to have access to the original text - written in English, in order to avoid any influence on their translation of the words. The same revisers assessed the three resulting versions, comparing them with the original document in English.

#### Cultural adaptation

The cultural adaptation of the Brazilian SADL aimed at establishing the cultural equivalence between the English and the Portuguese versions of the questionnaire. According to Guillemin et al. (1993)^14^, cultural equivalence is established when there are no understanding difficulties associated with the questions created or with the terms used by part of the study population, when at least 80% of the individuals did not report any type of difficulty to answer the question created. Should this number go beyond what was established, the questions are individually submitted to a new translation process. One first interviewer (interviewer 1) applied the questionnaire, reading out loud each question, in order to include those who had visual problems or were illiterate. Thirty patients fit with ISAD were individually interviewed.

#### Questionnaire reproducibility

In order to test inter-researcher reproducibility, the questionnaire was used with the same thirty interviewed patients in the phase of cultural adaptation by a second interviewer (interviewer 2). On the same day of the interview the questionnaire was employed again by the first interviewer (interviewer 1) in order to assess intraexaminer reproducibility.

#### Statistical method

In order to obtain the results from the mean, median, minimum and maximum values and standard deviation for each question on the questionnaire in both applications of it we used a descriptive statistical analysis. As far as reproducibility was concerned, we used the Spearman Coefficient Test and, to compare each application of the SADL we used the Wilcoxon test.

## RESULTS

In the present study, the mean age of the participants who answered the SADL was 66.36 years of age, ranging between 19 and 88 years. As to gender, we had 19 men (63%) and 11 women (37%).

The ISAD users who participated in this study showed a greater satisfaction level associated with questions: 1 (mean: 6.35), 3 (6.85), 5 (6.10), 6 (6.80), 8 (6.33), 9 (6.80), 11 (6.16), 12 (6.93) and 15 (6.52), while a lower satisfaction rate was found with the following questions 4 (3.16), 7 (3.41) and 13 (2.83). We compared each question in terms of the first and second questionnaire application and we did not observe statistically significant results (Table 1).

**Table 1:** Distribution of the mean value results between the first and the second application of each question in the SADL questionnaire as to the mean, media, minimum value, maximum value and standard deviation.

SADL question	N Subjects	Mean	Median	Minimum	Maximum	Standard Deviation
1	30	6.35	7.00	3.00	7.00	1.07
2	30	4.53	4.50	1.00	7.00	2.16
3	30	6.85	7.00	5.00	7.00	0.47
4	30	3.16	2.25	1.50	7.00	2.37
5	30	6.10	7.00	1.00	7.00	1.49
6	30	6.80	7.00	4.00	7.00	0.61
7	30	3.41	2.25	1.00	7.00	2.50
8	30	6.33	7.00	4.00	7.00	1.07
9	30	6.80	7.00	5.50	7.00	0.44
10	30	6.00	6.50	3.00	7.00	1.28
11	30	6.16	6.75	1.50	7.00	1.27
12	30	6.93	7.00	6.00	7.00	0.25
13	30	2.83	1.00	1.00	7.00	2.33
14	30	5.38	6.00	1.00	7.00	1.55
15	30	6.52	7.00	2.00	7.00	1.30

The results associated with the questionnaire subscales revealed a greater satisfaction associated with the Positive Effect (6.50) and Service and Costs (6.26) subscales. We observed a lower satisfaction level associated with the Negative Factors subscale (4.73) (Table 2).

**Table 2:** Distribution of the mean results between the first and the second application as to the SADL Questionnaire subscales.

SADL subscale	Mean	Median	Minimum	Maximum	Standard Deviation
Positive Effect	6.50	6.57	5.30	7.00	0.49
Services and Costs	6.26	6.70	3.10	7.00	0.93
Negative Factors	4.73	4.67	2.15	7.00	1.50
Personal Image	5.43	6.00	2.70	7.00	1.60
Global	5.88	5.85	4.80	7.00	0.67

These results indicated good questionnaire reproducibility.

The users were considerably pleased with their ISAD use.

## DISCUSSION

The hearing loss may be considered as one of the most disabling sensorial changes that affect human beings. Facing such change, we noticed a reduction in social contacts, yielding emotional disorders, which often times can be devastating for this population^15^. Moreover, this loss can cause difficulties for the individual to have an effective communication in the social and, especially, his occupational settings^16^.

Among the currently available technologies and resources, the ISAD cropped up as a means to minimize the hearing loss effects.

The Brazilian literature has some self-assessment questionnaires which were translated and adapted to our country, used to investigate the level of satisfaction, the benefits obtained from the use of ISAD and the reduction in auditory handicap with the use of amplification.

For this study we selected the SADL questionnaire in order to check its real distribution in the clinical practice, to assess the level of satisfaction of the individual users of ISAD and to culturally adapt the SADL in order to apply it to the Brazilian population.

The SADL tool has 15 questions broken down into four subscales: Positive Effects (6 items associated with the acoustic and psychological benefit), Services and Costs (3 items associated with professional competence, product price and number of repairs), Negative Factors (3 items associated with the amplification of environmental noise, the presence of feedback and its use on the telephone) and Personal Image (3 items associated with aesthetic factors and the very stigma of using ISAD) (Attachment I).

The mean age of the participants who answered the SADL was 66.36 years, ranging between 19 and 88 years of age. As far as gender is concerned, our study counted on 19 men (63%) and 11 women (37%).

ISAD users who participated in this study showed a greater user satisfaction associated with the following questions: 1 (mean: 6.35), 3 (mean: 6.85), 5 (mean: 6.10), 6 (mean: 6.80), 8 (mean: 6.33), 9 (mean: 6.80), 11 (mean: 6.16), 12 (mean: 6.93) and 15 (mean: 6,52). Considering less satisfaction, we found the following results: question 4 (mean: 3.16), 7 (mean: 3.41) and question 13 (mean: 2.83) (Table 1). We compared each question in association with the first and the second questionnaire application and we did not observe statistically significant results.

Results associated with the questionnaire subscales revealed a greater user satisfaction for the Positive Effect (mean: 6.50) and Services and Costs (mean: 6.26) and less satisfaction for the Negative Factors subscale (mean: 4.73) (Table 2). We did not find statistically significant results in comparing the SADL subscales.

Having these results, it is important to mention that to monitor patient satisfaction is fundamental to assess clinical procedures, to obtain quality assurance and because satisfaction reflects the true results of a health care initiative. As we identify the factors which may contribute to patient satisfaction and as we attempt to provide such attributes to the processes involved, we have the potential to obtain a more effective outcome at the health care services^17^.

Findings indicated a large user satisfaction for the Positive Effect subscale, and this fact is corroborated by other studies^7^,^8^,^18^,^19^. These authors reported that the Positive Effect has a strong influence on the satisfaction make up; and they also mentioned that this scale is important because of the fact that the improvement in communication and sound quality can be noticed early on when the patient starts using the ISAD and it is not much susceptible to changes along time. Nonetheless, one study reported that those individuals who expect more psychological and psychoacoustic benefits from their devices before fitting, tended to have a greater satisfaction in these aspects after fitting. Sometimes, individuals believe that the ISAD make the person look incompetent and this is a serious impediment to a good result in amplification, which is reinforced by the understanding that new users have lower expectations when compared to more experienced users^11^. This finding corroborates the study of authors^18^ who compared the satisfaction score of people who had been using their ISAD for two weeks with another group of users who had been using their ISAD for 12 months and concluded that ISAD patients who had been using their devices for less time are more satisfied. In relation to a recent study, in which the researchers^20^ assessed their capacity to use ISAD in experienced users, the results suggest that hearing device users of longer time had experience and an excellent understanding on how to use them.

ISAD users in this study did not complain of device repair, and they were very much pleased in relation to question 15 (mean: 6.57). (Table 1). Researchers^19^ have found the opposite, where question 15 was the one which showed dissatisfaction towards the ISAD, the authors concluded that it is possible that this question was considered a source of dissatisfaction because the users, participants in this study had been fitted for a very short time. They also noticed that the application of the SADL questionnaire is more efficient after one month of fitting.

In regards of the ISAD fitting time and SADL application, we found the use of the questionnaire after six weeks of fitting^13^. We also found it in literature studies which applied the SADL after three months of fitting^21^. In the present study we used as an inclusion criterion at least six months of use (54%) and 1 to 10 years of use (46%), according to the questionnaire's proposal.

We also noticed a high satisfaction rate in relation to the Services and Costs subscale, indicating that the users did not complain of acoustic issues, in relation to the audiologist's competence, and the quality of the ISAD (Table 2). In one given study^7^ the authors reported that question 10, about the natural trait of sounds perceived with the ISAD, was considered by the researchers as one of drawbacks of SADL. This happened because it is very difficult to assess individuals who had been using their hearing aids for a long time. With this, one can suppose that these users are not used to the sound transmitted by the ISAD and are unable to tell the difference between amplified and not amplified sounds. In the current study we did not find statistically significant differences in relation to question 10, although a satisfactory result was achieved because there was a mean score of 6.0 for satisfaction.

The items in the Negative Factors subscale which investigate the performance of users in a noisy environment, feedback and the use of telephone were listed as dissatisfactory by the users of hearing aid in the study and such domain was then created by the authors as a thermometer of fitting problems^7^ (Table 2).

As to speech understanding under noise, the authors^22^ reported that ISAD users in their study had satisfaction values with their hearing aids evaluated by means of the SADL questionnaire. They also reported that although speech understanding under noise or in situations of group meetings continues being problematic, the individuals reported use of their hearing aids almost all the time whether or not in easy or difficult hearing situations.

It was noticed that the difficulty in using the telephone was considered one of the most important complaints in the SADL questionnaire seen in the studies^8^,^23^,^24^. This complaint may happen because to speak on the phone is an auditory situation in which the ISAD technical limitations are made clear. For this reason, it is necessary to better educate the patients concerning the use and handling of their devices, which should be reinforced by training them to use it with the telephone and to educate also the professionals responsible for the fitting process. The speech and hearing therapist must stress that the inherent difficulties are unavoidable, so that the patient does not come up with false expectations followed by frustration^25^.

In regards of question four (4), which is associated with the hearing aid image and the stigma of hearing loss, it was considered non-satisfactory by the participants in this study. In a given study^8^ the Personal Image subscale was maintained by the authors based on the finding that for some individuals the appearance of the hearing aid and the impression they make on others is extremely significant, although many users were not concerned with such issues.

During our bibliographic survey on SADL, we noticed that this questionnaire was very useful in different situations in order to learn about the degree of satisfaction of individuals with hearing loss, since the literature showed its efficacy to check the satisfaction of mothers of children using ISADs^26^, in elderly^27^, in order to determine the degree of satisfaction according to the type and model of hearing aid^28^ and in users of cochlear implant^29^.

Moreover, some studies tell us that the SADL may be considered a gold standard evaluation tool to learn about the satisfaction of ISAD users^11^. Its practical scoring and numeric indices enable us to understand how the individual behaves in relation to a normative group. It was considered adequate to estimate the level of satisfaction because it is short, primordial to obtain subjective data, it is geared towards clinical use and it enables a subjective and independent measurement of the elements which make up satisfaction^10^, ^11^, ^12^, ^13^,^30^.

Finally, the benefits of hearing aids are associated with an improvement in communication in daily life, including the reduction in the auditory handicap of ISAD users. Results with the hearing aid go beyond its benefits, and satisfaction stands out as a more reliable measure of such results, because it encompasses a number of factors, it bears a dynamic character, and it depends on the user's perception and behavior, which is not associated only with the ISAD performance^7^,^8^,^25^.

## CONCLUSION

• We did not find statistically significant results among the questionnaire's subscales;
• Reproducibility was considered satisfactory;
• This study yielded a good performance to identify the questionnaire efficacy, because the users were considerably satisfied with the ISAD.

**Attachment 1**

**SATISFACTION WITH AMPLIFICATION IN DAILY LIFE ATISFACTION WITH AMPLIFICATION IN DAILY LIFE WITH AMPLIFICATION IN DAILY LIFE**

**Name ____________________ Date of Birth___/___/___ Today's Date___/___/___**

**INSTRUCTIONS**

**Listed below are questions on your opinions about your hearing aid(s). For each question, please circle the letter that is the best answer for you. The list of words on the right gives the meaning for each letter.**

**Keep in mind that your answers should show your general opinions about the hearing aids that you are wearing now or have most recently worn.**

**A Not At All**
**B A Little**
**C Somewhat**
**D Medium**
**E Considerably**

1 Compared to using no hearing aid at all, do your hearing aids help you understand the people you speak with most frequently?
  A B C D E F G
2 Are you frustrated when your hearing aids pick up sounds that keep you from hearing what you want to hear?
  A B C D E F G
3 Are you convinced that obtaining your hearing aids was in your best interests?
  A B C D E F G
4 Do you think people notice your hearing loss more when you wear your hearing aids?
  A B C D E F G
5 Do your hearing aids reduce the number of times you have to ask people to repeat?
  A B C D E F G
6 Do you think your hearing aids are worth the trouble?
  A B C D E F G
7 Are you bothered by an inability to get enough loudness from your hearing aids without feedback (whistling)?
  A B C D E F G
8 How content are you with the appearance of your hearing aids?
  A B C D E F G
9 Does wearing your hearing aids improve your self-confidence?
  A B C D E F G
10 How natural is the sound from your hearing aids?
  A B C D E F G
11 How helpful are your hearing aids on MOST telephones with NO amplifier or loudspeaker?
  (If you hear well on the telephone without hearing aids, check here [ ] )
  A B C D E F G
12 How competent was the person who provided you with your hearing aids?
  A B C D E F G
13 Do you think wearing your hearing aids makes you seem less capable?
  A B C D E F G
14 Does the cost of your hearing aids seem reasonable to you?
  A B C D E F G
15 How pleased are you with the dependability (how often they need repairs) of your hearing aids?
  A B C D E F G
  Please respond to these additional items.

**Table:** 

EXPERIENCE WITH CURRENT HEARING AIDS	LIFETIME HEARING AID EXPERIENCE (includes all old and current hearing aids)	DAILY HEARING AID USE	DEGREE OF HEARING DIFFICULTY (without wearing a hearing aid)
		None	
Less than 6 weeks	Less than 6 weeks	Less than 1 hour per day	None
6 weeks to 11 months	6 weeks to 11 months	1 to 4 hours per day	Mild
1 to 10 years	1 to 10 years	4 to 8 hours per day	Moderate
Over 10 years	Over 10 years	8 to 16 hours per day	Severe

For audiologists use only.

**Table:** 

	HEARING AID FITTING
Right Ear	Left Ear
Make	Make
Model	Model
Series Number	Series Number
Fitting Date	Fitting Date
Style: CIC ITC ITE BTE	Style: CIC ITC ITE BTE
	HEARING AID FEATURES (check all that apply)

Directional Microphones T-coil
Multiple Microphones Peak Clipping
Multi-channel Compression Liming
Continues in Attachment 1
Remote Control TILL
Multi-program WDRC
No volume control BILL
Other

**Attachment 2**

**SATISFAÇÃO COM AMPLIFICAÇÃO NA VIDA DIÁRIA**

**Nome: _____________________ Data de Nascimento __/__/__Data de Hoje __/__/__**

**INSTRUÇÕES**

**As questões listadas abaixo se referem as suas opiniões sobre o seu aparelho auditivo.**

**Para cada questão, por favor, circule a letra que responde melhor a questão para você. A lista à direita fornece o significado de cada letra.**

**Lembre-se que suas respostas devem mostrar suas opiniões gerais sobre o aparelho auditivo que você está usando atualmente ou que tem usado mais recentemente.**

**A Não**
**B Muito pouco**
**C Pouco**
**D Médio**
**E Ás vezes**
**F Quase sempre**
**G Sempre**

1 O seu aparelho auditivo lhe ajuda a entender as pessoas com as quais você fala quando comparado a época que não usava aparelho auditivo?
  A B C D E F G
2 Você fica frustrado quando o seu aparelho auditivo capta sons que lhe impedem de ouvir o que você quer?
  A B C D E F G
3 Você está convencido de que a obtenção de seu aparelho auditivo fazia parte dos seus maiores interesses?
  A B C D E F G
4 Você acha que as pessoas percebem mais a sua perda auditiva quando você usa o aparelho?
  A B C D E F G
5 O seu aparelho auditivo reduz o número de vezes que você tem que pedir para as pessoas repetirem?
  A B C D E F G
6 Você acha que vale a pena usar o aparelho auditivo?
  A B C D E F G
7 Você se sente incomodado quando necessita aumentar o volume e ocorre a microfonia?
  A B C D E F G
8 Você está contente com a aparência do seu aparelho auditivo?
  A B C D E F G
9 O uso do seu aparelho auditivo melhora a sua autoconfiança?
  A B C D E F G
10 O som do seu aparelho auditivo é natural?
  A B C D E F G
11 O seu aparelho auditivo é útil na MAIORIA dos telefones sem amplificador ou caixas de som?
  A B C D E F G
  (Se você ouve bem ao telefone sem o aparelho, marque aqui )
12 A pessoa que lhe forneceu o aparelho auditivo era competente?
  A B C D E F G
13 Você acha que usar o aparelho lhe faz parecer menos capacitado?
  A B C D E F G
14 O custo do seu aparelho auditivo parece razoável para você?
  A B C D E F G
15 Você está satisfeito com a frequência com a qual seu aparelho auditivo precisa de reparos?
  A B C D E F G

Por favor, responda os itens adicionais.

**Table:** 

EXPERIÊNCIA COM O APARELHO ATUAL	EXPERIÊNCIA COM O APARELHO POR TODA VIDA (incluindo todos os aparelhos que já usou)	USO DIÁRIO DO APARELHO AUDITIVO	GRAU DE DIFICULDADE AUDITIVA (sem o aparelho)
		Nenhum	
Menos de 6 semanas	Menos de 6 semanas	Menos de 1 hora por dia	Nenhum
De 6 semanas a 11 meses	De 6 semanas a 11 meses	1 a 4 horas por dia	Leve
De 1 a 10 anos	De 1 a 10 anos	4 a 8 horas por dia	Moderada
Mais de 10 anos	Mais de 10 anos	8 a 16 horas por dia	Severa
USO EXCLUSIVO DOS FONOAUDIÓLOGOS			

	ADAPTAÇÃO DO APARELHO
Orelha direito	Orelha Esquerdo
Fabricação ______________________	Fabricação ______________________
Modelo _________________________	Modelo _________________________
N° série ________________________	N° série ________________________
Data da adaptação ________________	Data da adaptação _______________
Estilo CIC ITC ITE BTE	Estilo CIC ITC ITE BTE
CARACTERÍSTICAS DO APARELHO (marque todos que se aplicarem)

Microfone Direcional Peak Clipping
Microfones Múltiplos Limitação por Compressão
Multicanal TILL
Controle Remoto WDRC
Multiprograma BILL
Sem controle de volume T-Coil
Outros
